# Comprehensive Evaluation of the Utility of Cell Block Use in ThinPrep Liquid-Based Cervical Specimens

**DOI:** 10.5146/tjpath.2025.13802

**Published:** 2025-09-30

**Authors:** Yasemin Akça, Evren Uzun, Suna Erkılıç

**Affiliations:** Department of Pathology, Gaziantep University, Faculty of Medicine, Gaziantep, Türkiye

**Keywords:** Cell-block, Thin Prep, Liquid-based cytology, Atrophic vaginitis, Epithelial cell abnormality

## Abstract

***Objective: *** The Papanicolaou (PAP) smear remains the cornerstone of early detection and prevention in cervical cancer screening, Today, liquid-based cytology (LBC) techniques are more widely used for this purpose. ThinPrep is one of the most effective of these methods. In this study, we aimed to investigate the contributions of the cell block method when using ThinPrep liquid-based cervical samples.

***Material and Methods:*** We retrospectively reviewed a total of 453 cases in which we applied cell block to assist in correct diagnosis in terms of four criteria we determined from ThinPrep LBC samples accepted to our department between 2020 and 2023. We investigated the benefits of cell block according to the four criteria we defined in these cases; these criteria were adequacy, determination of cellular origin based on atrophy, and correct diagnosis of squamous cell lesions and glandular cell lesions. Cell blocks were re-evaluated by 3 experienced pathologists, and the results were analyzed.

***Results: *** The cell block method contributed significantly to the adequacy in 97 of the 136 samples. It contributed to understanding the cellular origin and correct diagnosis of atrophic background in 113 of the 165 samples. It also contributed to the correct diagnosis of squamous cell lesions in 26 of the 107 samples and glandular cell lesions in 40 of the 45 samples. Overall, it was detected to be useful in 272 out of 453 cases.

***Conclusion:*** The cell-block method represents powerful contributions for each parameter, especially if it is used selectively, particularly in evaluating glandular cell lesions and atrophic background. Additionally, it facilitates ancillary testing in the field of cervical cancer screening and management.

## INTRODUCTION

In cervical cancer screening, the Papanicolaou smear remains the cornerstone of early detection and prevention. For many years, pathologists and scientists have desired to increase the sensitivity and specificity of the conventional Pap smear and have improved the liquid-based cytology (LBC) methods and the automated devices for both preparing the slides and screening. LBC resulted in complete and standardized cell transfer with monolayer cell preparation, decreased obscuring factors, improved fixation, and reduced screening time.

Until now, there have been two main methods of preparation - ThinPrep and SurePath. In 1996, the FDA approved ThinPrep as an alternative to the cervicovaginal smear in the United States. Approval for Autocyte Prep (also called SurePath or CytoRich Prep) followed in 1999 (1).

With the progress of liquid-based technologies and immunohistochemical and molecular techniques in the differential diagnosis, the cell block method has entered our lives and is widely used now. Although it is widely used for pleural and peritoneal exfoliative cytology and lymph nodes, this is not the case for cervical PAP smears.

In addition, although we have identified many important and influential articles in the literature, particularly on lung, pleural, and lymph node liquid-based cytology specimens, we have found a few articles on cervical PAP smears. The majority of these articles focused on specific aspects of their utility. In the present study, we aimed to explore the comprehensive utility of cell block use in terms of all parameters while evaluating a cervicovaginal smear specimen, including immunohistochemical examination when necessary.

## MATERIAL and METHODS

### Sample Selection

We retrospectively reviewed a total of 453 cases in which we applied cell block to assist in the correct diagnosis in terms of four criteria we determined from ThinPrep LBC samples accepted to our department between 2020 and 2023. We investigated the benefits of cell block according to the 4 criteria we defined in these cases;

These criteria were,

- Cellular adequacy

- Determination of the cellular origin on the background of the atrophy

- Squamous cell lesions

- Glandular cell lesions

We first determined cell block was applied to 136 of the cases for adequacy, 165 of the cases for atrophic background, 107 of the cases for squamous cell lesions, and 45 of the cases for glandular cell lesions. Although some cases could be evaluated according to more than one criterion, to avoid confusion, each selected group was evaluated according to its own criteria.

### While Preparing Cell Block

We have used the ThinPrep® liquid-based cytologic system for cytological specimens in our laboratory for 10 years. After preparing the slides from the specimen, we prepared cell blocks from residual material. The cell block was prepared by first centrifuging the remaining liquid at 4000 rpm for 4 minutes after slide preparation, then adding 3 ml of 96 % ethyl alcohol and 0.5 ml of 10% formaldehyde to the bottom sediment, waiting for 60 minutes, and collecting the remaining sediment on filter paper. It was then placed in a cassette and kept in 96% ethyl alcohol until placed in the tissue processing device. Finally, it was processed starting from the alcohol stage in the tissue processing device.

### Evaluation

Three experienced pathologists reevaluated the slides, first without and then with the cell block, noting the contribution of the cell block to the correct diagnosis according to ‘’The Bethesda System for Reporting Cervical Cytology, third edition’’ (2) established criteria. Various immunohistochemical stains were also used on the cell block sections if they were needed to clarify the diagnosis.

## RESULTS

A total for 136 cases that were unsatisfactory for evaluation due to unsatisfactory squamous cellularity and other quality indicators such as partially obscuring blood or severe inflammation were included in the study. The presence of endocervical cells was also assessed. Fifty-six had low squamous cellularity and no endocervical cells on smear evaluation and 30 had limitations to evaluate due to inflammation and bloody background. Forty had adequate squamous cellularity but no endocervical cells. Ten had endocervical cells but low squamous cellularity. We observed a significant increase in cellularity in 97 samples. Seventy-nine of these samples showed a significant increase in squamous cell count, 8 of these showed the presence of endocervical cells not observed in the smear, and 10 of these showed both an increase in squamous cellularity and the presence of endocervical cells. In 39 cases we have not found any contribution.

A total of 165 atrophic cases with or without inflammation were included in the study and we found useful contributions in 113 cases. It provided the correct diagnosis of epithelial abnormalities in 8 squamous, 4 glandular, and a total of 12 cases. It increased the number of squamous cells in 30 cases and clarified the suspected epithelial origin in 26 cases. In 12 cases it helped us to correctly identify basal-parabasal cells on atrophic ground which could be misinterpreted as atypical glandular cells or atypical squamous cells. In 33 cases, it supported us to differentiate between endocervical and endometrial cell origin.

Finally, 107 samples were included for the correct diagnosis of squamous cell lesions and made useful contributions in 26 cases. Similarly, it made useful contributions in 40 of the 45 samples in diagnosing glandular cell lesions. At this point, it should be emphasized that we used some immunohistochemical markers to make the correct diagnosis. They were especially useful in determining the origin of atypical cells. The results are detailed in Table 1 and Table 2.

**Table 1:** Contributions of cell block in squamous cell lesions and case numbers

Total number of cases with benefits	26		26
Number of cases transferred to a higher category	ASCUSàLSIL	5	14
	ASCUSàHSIL	1	
	UnsatisfactoryàHSIL	4	
	LSILàHSIL	3	
	ASC-HàHSIL	1	
Number of cases supporting the diagnosis	LSILàLSIL	7	12
	HSILàHSIL	5	

**ASCUS: **Atypical squamous cells of undetermined significance, **ASC-H:** Atypical squamous cells cannot exclude an HSIL, **LSIL:** Low–grade squamous intraepithelial lesion, **HSIL: **High–grade squamous intraepithelial lesions.

**Table 2:** Contributions of cell block in glandular cell lesions and case numbers

Total number of cases with benefits	40		40
The number of cases transferred to a higher category	Atypical endometrial cells, NOSàEndometrial Adenocarcinoma	4	6
	Atypical endocervical cells, NOSàEndocervical Adenocarcinoma	2	
The number of cases transferred to a lower category which cannot definitively determine its nature in smear samples	Glandular Cell, NOSàNegative for malignancy	34	34

**AGC: **Atypical glandular cells, **NOS: **Not otherwise specified

Overall, the cell block method was observed to be quite useful in 272 out of 453 cases and the details are summarized in Table 3.

**Table 3:** Summary of the contributions of the cell block for each parameter

**Parameters**	**Total number of cases**	**Number of cases contributed**	**Percentages**
Adequacy	136	97	71.3%
Atrophic background	165	113	68.4%
Squamous cell lesions	107	26	24.2%
Glandular cell lesions	45	40	88.8%
Total number	453	276	60.9%

## DISCUSSION

LBC is widely used for preparing gynecological and nongynecological cytology specimens in the daily pathology laboratory routine. LBC preparations allow cell enrichment through the reduction of background inflammatory and blood cells. The presentation of cells in a uniform layer through an automated and standardized process enhances the identification of malignant cells. In addition, residual specimens can be used in immunocytochemical and molecular studies (3).

Although LBC is useful, we usually encounter some problems while evaluating the slides. One of the major problems is cellular adequacy. Despite the superiority of the liquid-based technique in this regard, both the inflammatory background and blood cells may prevent us from evaluating the cells. In addition, samples with poor cellular adequacy are another problem. We determined additional contributions in 97 of the 136 cases, and this ratio could not be ignored. Giving the chance to evaluate the same sample by using an additional easy method will be more effective both financially and for the patient than resampling.

**Figure 1:**
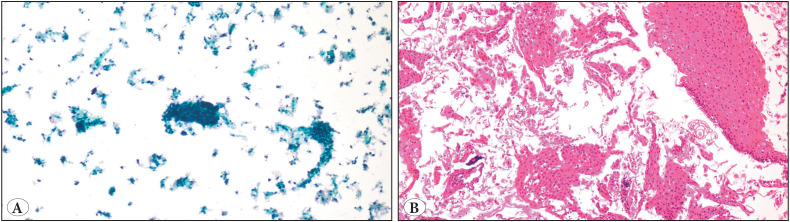
**A)** X100 magnification of atypical appearing cells in an atrophic and bloody background in a PAP Smear sample. The cellular origin could not be easily detected. **B)** X100 magnification. Examples of cervicovaginal epithelium showing atrophy that has fallen into compact layers in the cell-block section of the smear sample in picture A

**Figure 2:**
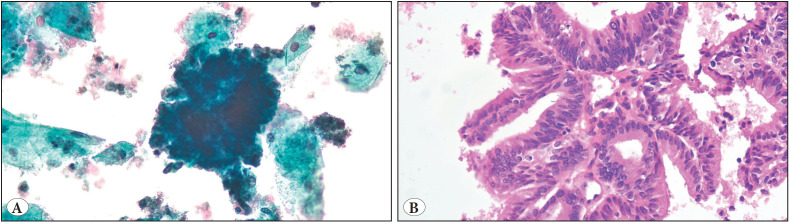
**A)** X400 magnification of atypical glandular cells in a bloody background in a PAP Smear sample. **B)** X200 magnification of endometrioid adenocarcinoma in a cell-block section of the smear sample in Picture A. We can easily notice the villoglandular and cribriform patterns of the atypical cells.

**Figure 3:**
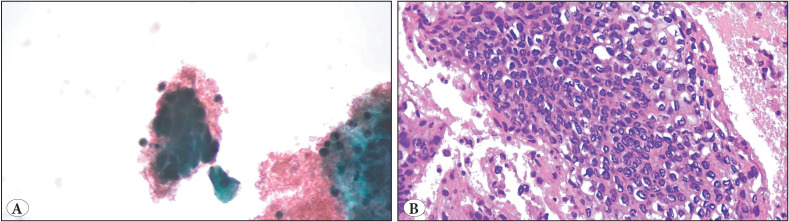
**A)** x400 magnification of a hyperchromatic crowded group in an atrophic and bloody background in a PAP Smear sample. **B)** x400 magnification of high-grade squamous intraepithelial lesion on a cell-block section of the smear sample in Picture A. We could see the atypical changes through full-thickness

Another issue is that it is not always easy to determine the cellular origin, especially in cases of intense inflammation, hemorrhage, and reactive changes. Also, one of the major problems is atrophic background in LBC in this regard. In a study, ThinPrep atrophic vaginitis slides were significantly more likely to be deemed unsatisfactory. In that study, some participants confused HSIL with atrophic vaginitis, most often on ThinPrep slides and least often on SurePath slides. They hypothesized this may be due to the increased tendency of groups in ThinPrep slides to round up, overlap, and shrink, making evaluation of cell size and individual nuclei more difficult. Additionally, both atrophic parabasal cells and HSIL involving endocervical glands show the presence of inconspicuous nucleoli. Some participants could interpret hyperchromatic, crowded, atrophic parabasal cells as the hyperchromatic, crowded groups of HSIL (4). In this regard, Renshaw et al. demonstrated that HSIL in CAP educational ThinPrep Pap slides is mistaken for a glandular lesion by 75% of expert cytopathologists from the Cytopathology Resource Committee in another study (5). In some cell block sections, we noticed that atypical cells fell in typical tumoral patterns or in sheets or microtissues, which made the diagnosis much easier (Figure 1, Figure 2, Figure 3).

In addition, we need to apply immunohistochemical staining to accurately determine the degree of dysplasia, find the tumor subtype, and distinguish possible benign conditions from malignancy as pathologists. Although we can apply some immunohistochemical markers on LBC preparations, immunostained cytology preparations would not allow coordinated evaluation of multiple immunomarkers on the same cells in a fashion comparable to that achieved in serial sections of the cell block (6). Also, it was depicted that immunostaining of the sections of cell block from residual cervical LBC would increase the positive predictive value for the detection of HSIL and higher lesions in sections of cell block from residual LBC specimens equivalent to brush biopsy, with the results comparable to surgical biopsies in another study (7). To be able to perform immunohistochemical staining with a wide range safely is an excellent advantage.

Another advantage is in the molecular area. Application of high-throughput techniques to biological samples such as cervical cytology specimens, including the cell blocks prepared from it, allows analysis of clusters of differential molecular profiles related to the genome, the transcriptome, or the proteome. This analysis may generate molecular signatures of a particular cancer, including different grades of CIN and cervical cancer. In addition to diagnostic and therapeutic contributions, it also assists in elucidation of the pathways related to the development and progression of cancer. This may assist in detecting cervical cancer in its early stages, with improvement in survival rate, prognosis, and recurrence (8).

Cell blocks allow various molecular pathology tests, including gene rearrangement studies and FISH, proteomics analysis, and microbiology/histochemical special stains for hematolymphoid lesions (9). Similarly, LBC became an important source of the molecular characterization of non-small cell lung carcinomas (10). Likewise, the ability to safely perform genetic tests on cervical cytological material provides avoiding more invasive biopsy methods and their complications, especially in unresectable tumors.

Various studies indicate that cell blocks increase diagnostic accuracy from different perspectives, which is in line with the content. In one study, Kuzucular et al. revealed that the cell block was superior to the LBC method in terms of diagnostic accuracy for the detection of HSIL (11). Shidham et al. underlined that the value of making a cell-block from a Pap test sample is to uncover cellular architecture, or perform immunohistochemistry, to help reach a definitive diagnosis in challenging cases (12). Akpolat et al. emphasized that the most noteworthy findings of their study are that cell block analysis can be used to distinguish HSIL from atrophy and metaplasia and it may be possible to analyze HPV DNA–positive ThinPrep specimens from high-risk patients using p16INK4a immunohistochemistry so that the detection of CIN2/CIN3 is increased and patient recall is minimized (13).

The importance of the hyperchromatic crowded groups (HCGs) is discussed in some articles (14,15). We noticed that Pap test cell-blocks are useful to clarify the diagnosis of HCG’s, distinguish glandular from squamous lesions, separate immature squamous metaplasia from high grade squamous intra-epithelial lesions (HSIL), discriminate repair from squamous cell carcinoma (SCC), and discern tubal metaplasia from atypical glandular cells (AGCs) in our study.

PAP smear is a screening method, and therefore routine application of CB to all cervical PAP smear samples may not be cost-effective, however, in our study, we found significant contributions in all aspects of the evaluation criteria. Furthermore, this is a comprehensive study in line with the previous literature, so we can claim that it is a promising strategy to maximize the effectiveness of PAP smear-based screening programs and ultimately reduce the burden of cervical cancer worldwide.

## CONCLUSION

The cell block method represents a powerful contribution to analysis for each parameter, especially when used selectively, offering enhanced diagnostic accuracy, additionally facilitating ancillary testing, and expanding research opportunities in the field of cervical cancer screening and management.

## Conflict of Interest

The authors declare no conflict of interest

## References

[ref-1] Makde Manjiri Milind, Sathawane Prajakta (2022). Liquid-based cytology: Technical aspects. Cytojournal.

[ref-2] Nayar R, Wilbur DC (2015). The Bethesda System for Reporting Cervical Cytology: Definitions, Criteria, and Explanatory Notes.

[ref-3] Moriarty Ann T., Schwartz Mary R., Ducatman Barbara S., Booth Christine N., Haja Jennifer, Chakraborty Subhendu, Williamson Beth, College of American Pathologists (2008). A liquid concept--do classic preparations of body cavity fluid perform differently than ThinPrep cases? Observations from the College of American Pathologists Interlaboratory Comparison Program in Nongynecologic Cytology. Arch Pathol Lab Med.

[ref-4] Crothers Barbara A., Booth Christine N., Darragh Teresa M., Means Marilee M., Souers Rhona J., Thomas Nicole, Moriarty Ann T. (2012). Atrophic vaginitis: concordance and interpretation of slides in the College of American Pathologists Cervicovaginal Interlaboratory Comparison Program in Gynecologic Cytopathology. Arch Pathol Lab Med.

[ref-5] Renshaw Andrew A., Mody Dina R., Wang Edward, Haja Jennifer, Colgan Terence J., Cytopathology Resource Committee, College of American Pathologists (2006). Hyperchromatic crowded groups in cervical cytology--differing appearances and interpretations in conventional and ThinPrep preparations: a study from the College of American Pathologists Interlaboratory Comparison Program in Cervicovaginal Cytology. Arch Pathol Lab Med.

[ref-6] Shidham Vinod B., Layfield Lester J. (2021). Cell-blocks and immunohistochemistry. Cytojournal.

[ref-7] Shidham Vinod B. (2022). Role of immunocytochemistry in cervical cancer screening. Cytojournal.

[ref-8] Martínez-Rodríguez Fátima, Limones-González Jared E., Mendoza-Almanza Brenda, Esparza-Ibarra Edgar L., Gallegos-Flores Perla I., Ayala-Luján Jorge L., Godina-González Susana, Salinas Eva, Mendoza-Almanza Gretel (2021). Understanding Cervical Cancer through Proteomics. Cells.

[ref-9] Alrajjal Ahmed, Choudhury Moumita, Yang Jay, Gabali Ali (2021). Cell-blocks and hematolymphoid lesions. Cytojournal.

[ref-10] Akca Yasemin, Erkilic Suna (2024). Methodological and TNM Focus-Based Comparison of EGFR Mutation Status in Non-Small-Cell Lung Carcinomas. J Cytol.

[ref-11] Kuzucular Elif, Ozden Ferhat, Muezzinoglu Bahar (2024). Comparison of liquid-based cytology and cell blocks prepared from cell remnants for diagnosis of cervical pathology. Ann Diagn Pathol.

[ref-12] Shidham Vinod B., Mehrotra Ravi, Varsegi George, D'Amore Krista L., Hunt Bryan, Narayan Raj (2011). p16 immunocytochemistry on cell blocks as an adjunct to cervical cytology: Potential reflex testing on specially prepared cell blocks from residual liquid-based cytology specimens. Cytojournal.

[ref-13] Akpolat Ilkser, Smith Debora A., Ramzy Ibrahim, Chirala Minni, Mody Dina R. (2004). The utility of p16INK4a and Ki-67 staining on cell blocks prepared from residual thin-layer cervicovaginal material. Cancer.

[ref-14] Chivukula Mamatha, Austin R. Marshall, Shidham Vinod B. (2007). Evaluation and significance of hyperchromatic crowded groups (HCG) in liquid-based paps. Cytojournal.

[ref-15] Abada Evi, George Kathleen, Shidham Vinod (2020). Hyperchromatic-crowded groups (HCG) in pap smears. Cytojournal.

